# Streptococcus constellatus Left Ventricular Apical Mural Infective Endocarditis With Acute Stroke, Septic and Cardiogenic Shock

**DOI:** 10.7759/cureus.22238

**Published:** 2022-02-15

**Authors:** Eric Hilker, Sachin M Patil, Zach Holliday, Niraj Arora

**Affiliations:** 1 Department of Medicine, University of Missouri School of Medicine, Columbia, USA; 2 Infectious Disease, University of Missouri, Columbia, USA; 3 Department of Medicine, Division of Pulmonary and Critical Care, University of Missouri School of Medicine, Columbia, USA; 4 Neurology, University of Missouri, Columbia, USA

**Keywords:** bacteremia, mural vegetation, stroke, infective endocarditis, streptococcus constellatus

## Abstract

Infective endocarditis (IE) is a severe infection of the endocardium and cardiac valves by multiple etiologic agents. Clinical presentation can be acute or subacute based on the host immunity and the causative agent’s virulence. Although *Streptococci* are responsible for most community-acquired native valve bacterial IE, *Streptococcus* *constellatus* is an infrequent cause. *S. constellatus* can rarely infect prosthetic cardiac valves. A middle-aged white male with poorly controlled type 2 diabetes mellitus was transferred to our facility for suspected stroke with an initial presentation of acute encephalopathy of uncertain duration. Transthoracic echocardiogram revealed a left ventricular apical mural vegetation, and brain imaging displayed multiple white matter hypodensities indicative of numerous small strokes. Blood cultures were positive for *S. constellatus*. Clinical presentation was unusual with an acute encephalopathy due to multiple septic emboli and primary mural IE with high-grade bacteremia due to *S. constellatus*. PubMed medical literature review reveals this to be a rare clinical presentation by an uncommon etiological agent with an infrequent echocardiogram finding.

## Introduction

Infective endocarditis (IE) poses diagnostic and management difficulties. About 10,000 to 20,000 new cases occur per year in the United States of America [1]. IE is a critical public health and hospital issue with poor outcomes as the risk factors remain opaque [2]. Advanced diagnostic processes reveal a growing concern for an increased prevalence of diverse causative agents [2]. The most common causes of community-acquired native valve IE are *Streptococci viridans* species and *Staphylococcus aureus* species in healthcare-acquired native valve IE [3]. Among the *S. viridans* group, *S. constellatus* is an infrequent cause of IE and a part of the *S. milleri* group, including *S. intermedius* and *S. anginosus* [4,5]. They are commensals in the gastrointestinal and urogenital tract, and *S. constellatus* frequently colonizes the pharynx [4]. Compared to* S. viridans* other members, the *S. milleri* is distinguished by its aggressive clinical behavior causing invasive purulent infections in the central nervous system, gastrointestinal tract, liver, lung, and dental areas [6,7]. Here, we present a Caucasian male with poorly controlled diabetes mellitus presenting with acute encephalopathy due to multiple septic emboli and primary mural IE due to *S. constellatus* bacteremia.

## Case presentation

A 54-year-old male with a past medical history of hypertension, hyperlipidemia, type 2 diabetes mellitus, intravenous (IV) drug abuse, Fournier’s gangrene, and recurrent methicillin-resistant *Staphylococcal* *aureus* (MRSA) infections presented to an outside hospital with an acute altered mental state and stroke concern. He was last seen 48 hours prior to this. Home medications included metformin, detemir insulin, amphetamine/dextroamphetamine, pioglitazone, Lipitor, and dapagliflozin. Clinical evaluation revealed stable vitals other than a tachycardia of 137 beats per minute (bpm), a National Institute of Health Stroke Scale (NIHSS) of 15 with right-sided weakness, and acute encephalopathy. A 12-lead electrocardiogram revealed atrial fibrillation (120 bpm) and was initiated on diltiazem drip.

Laboratory work (Table 1) revealed leukocytosis, thrombocytopenia, elevated serum creatinine, anion gap, aspartate aminotransferase (AST), alanine transaminase (ALT), and hypoalbuminemia. IV insulin and fluids were administered for diabetic ketoacidosis (DKA).

**Table 1:** Laboratory values of tests done at the outside hospital.

1) White cell count	13,800/mL with bands of 8%, neutrophils 78%
2) Platelet count	60,000/mL
3) AST (aspartate aminotransferase)	49 U/L (5–34)
4) ALT (alanine transaminase)	199 U/L (0–55)
5) Albumin	2.6 g/dL (3.5–5)
6) Total bilirubin	2.3 mg/dL (0.2–1.2)
7) Creatinine	1.43 mg/dL (0.72–1.25)
8) Anion gap corrected	26
9) Lipase	16 U/L (8–78)
10) Creatinine kinase	104 U/L (30–200)
11) Serum ketones	Present (moderate amount)
12) Lactic acid	3.8 mmol/L (0.5–2.2)
13) Troponin I	0.414 ng/mL (0.00–0.50)
14) Alcohol level	<10 mg/dL (0–10)
15) Serum salicylate level	<0 mg/dL (0–30)
16) Blood glucose level	536 mg/dL
17) Urine analysis	Negative for urinary tract infection and proteinuria

Computed tomography (CT) of the head plain revealed no acute intracranial abnormality. Empirically, he was given IV clindamycin for suspected sepsis with a history of prior MRSA infections. He was diagnosed with DKA, acute kidney injury, acute encephalopathy with suspected stroke, and sepsis. Due to his unknown duration of encephalopathy, fibrinolytic was not given, and he was transferred to our institution for acute stroke management.

On arrival at our institution, the patient was admitted to the neuro-intensive care unit for further stroke workup. Clinical examination revealed tachycardia (120 bpm), tachypnea (26/minute), worsening respiratory distress, NIHSS of 19 with right-sided weakness, slurry speech, somnolence, and unable to follow commands. Subsequently, he was intubated for acute respiratory failure and placed on mechanical ventilator support with sedation and analgesic medications. CT head, CT perfusion (CTP) of the head and CT angiogram (CTA) of the head and neck revealed bilateral centrum semiovale hypodensities, possibly representing chronic lacunar infarcts with no perfusion defect and 80% high-grade stenosis of the proximal right internal carotid artery with distal flow present. Chest X-ray portable revealed endotracheal tube and orogastric tube at the appropriate position with no acute cardiopulmonary abnormality.

Labs (Table 2) revealed leukocytosis (5% bands), thrombocytopenia, elevated internalized normalized ratio (INR), anion gap, lactic acid, beta-hydroxybutyrate, procalcitonin, troponin T, hemoglobin A1c, and creatinine.

**Table 2:** Laboratory values of tests done at our Institution. PCR: polymerase chain reaction; COVID-19: coronavirus disease 2019

Hospitalization day	Day 1	Day 2	Day 3
1) White cell count (3,500–10,500/mL)	22,100 (Bands 5%)	21,200	26,500 (Bands 1%)
2) Platelet count (150,000–450,000/mL)	61,000	53,000	96,000
3) International normalized ratio (0.9–1.1)	1.7	-	-
4) AST (aspartate aminotransferase) (0–40 U/L)	44	>7,000	>7,000
5) ALT (alanine transaminase) (10–50 U/L)	181	5,642	5,774
6) Total bilirubin (0.00–1.60 mg/dL)	1.66	2.34	3.03
7) Creatinine (0.70–1.20 mg/dL)	1.76	2.52	2.8
8) Anion gap (< 12 mmol/L)	21	14	<12
9) Beta-hydroxybutyrate (0.10–0.27 mmol/L)	4.12		
9) Ammonia (16–60 mcmol/L)	-	107	47
10) Erythrocyte sedimentation rate (ESR) (0.0–20 mm/h)	<1	<1	-
11) C-reactive protein (0.00–0.50 mg/dL)	8.26	6.02	11.51
12) Procalcitonin (0.00–0.05 ng/mL)	29.2	26.9	84.80
13) Lactic acid (0.5–2.2 mmol/L)	6.5	3.7	4.4
14) Troponin T (< 22 ng/L)	217	367	359
15) Creatinine kinase level (20–200 units/L)	493	265	208
16) Hemoglobin A1c (4–6%)	11.4%		
17) Thyroid stimulating hormone level (0.27–4.20 mcunit/mL)	-	8.280	
18) Volatile alcohol screen	-	negative	
19) Respiratory pathogen PCR panel	-	negative	
20) COVID-19 PCR test	negative		
21) Human immunodeficiency virus serology	negative		
23) Acute viral hepatitis panel	-	negative	

A comprehensive drug screen was positive for amphetamine, methamphetamine (Adderall use), metoprolol, diltiazem, and lidocaine. DKA was treated with IV fluids and insulin drip with a decline in anion gap. Broad-spectrum antibiotics IV vancomycin and aztreonam (penicillin allergy) were started for suspected sepsis. A transthoracic echocardiogram (TTE) with bubble study was ordered to rule out any thromboembolism and IE. Later in the day patient became hypotensive, requiring phenylephrine, and the diltiazem infusion was changed to amiodarone. The patient was successfully cardioverted to sinus rhythm with a synchronized shock of 200 joules by the cardiology team due to atrial fibrillation with hypotension. He was initiated on a heparin drip post-cardioversion to prevent any thromboembolic episodes. The clinical picture indicated acute sepsis rather than a stroke, and he was transferred to the medical intensive care unit.

**Figure 1:**
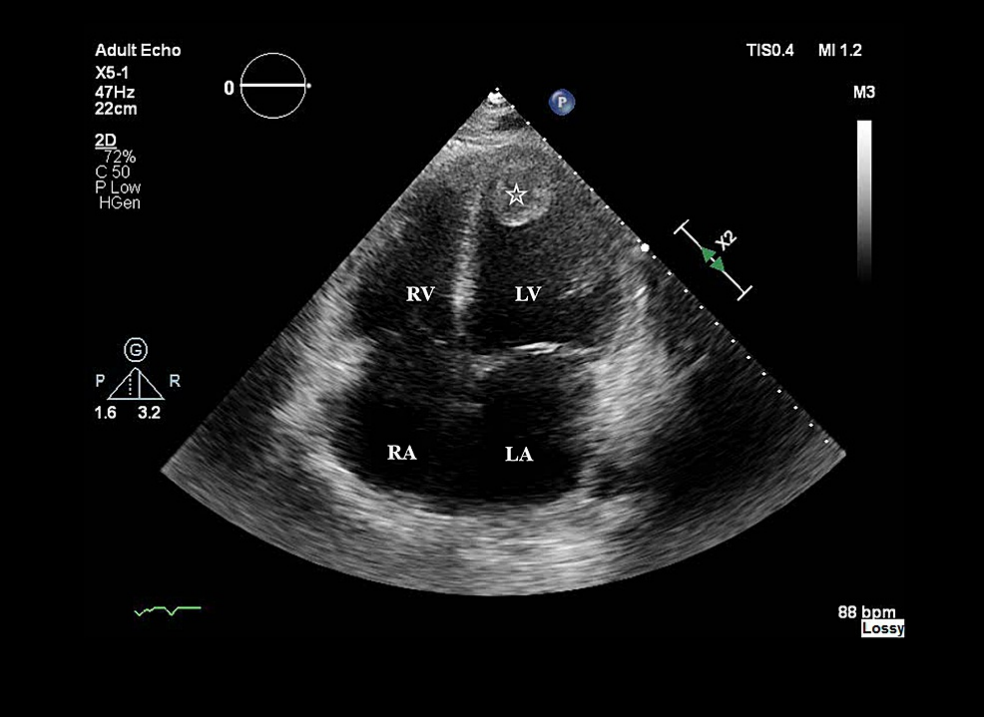
TTE apical view reveals the presence of an organized mass of 3 cm × 2.7 cm (White star) attached to the left ventricular apex. TTE: transthoracic echocardiogram

On day 2, bedside TTE revealed a low ejection fraction (EF), and dobutamine was started. He was initiated on hydrocortisone for refractory shock (five vasopressors: dobutamine, norepinephrine, phenylephrine, epinephrine, and vasopressin) and was febrile at 39.6°C. Labs (Table 2) revealed leukocytosis, thrombocytopenia, resolving anion gap, elevated AST, ALT, ammonia, lactic acid, procalcitonin, troponin, and inflammatory markers except for ESR. Acute viral hepatitis panel, volatile alcohol screen, and respiratory pathogen panel were negative. Abdominal ultrasound revealed mild hepatomegaly. TTE revealed severely increased left ventricle size with akinesia of the apical, anterior/distal walls and EF of 15%. It also revealed the presence of an organized mass of 3 cm × 2.7 cm attached to the left ventricular apex and an absence of atrial septal defect and valvular vegetations (Figure 1).

Due to minimal urine output patient was initiated on hemodialysis. Aztreonam was changed to meropenem. Later in the day, blood cultures at the outside hospital and our institution returned positive for Gram-positive cocci, and a transesophageal echocardiogram (TEE) was ordered. He was continued on IV heparin, insulin, and amiodarone drip. Due to his worsening clinical status, he was made a do not resuscitate after discussion with the family. On day 3, he continued to be in sinus tachycardia on five vasopressors and antibiotics. Labs revealed leukocytosis with bands of 6%, thrombocytopenia, worsening creatinine, elevated liver enzymes, lactic acid, procalcitonin, and troponin T (Table 2).

**Figure 2:**
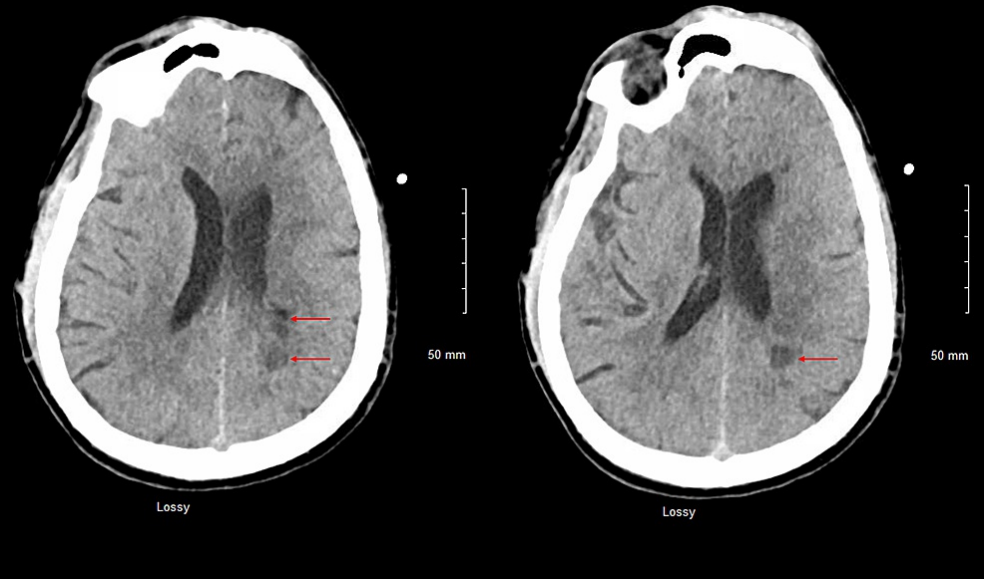
CT head axial view plain revealed multiple white matter hypodensities concerning septic emboli (Red arrows). CT: computed tomography

**Figure 3:**
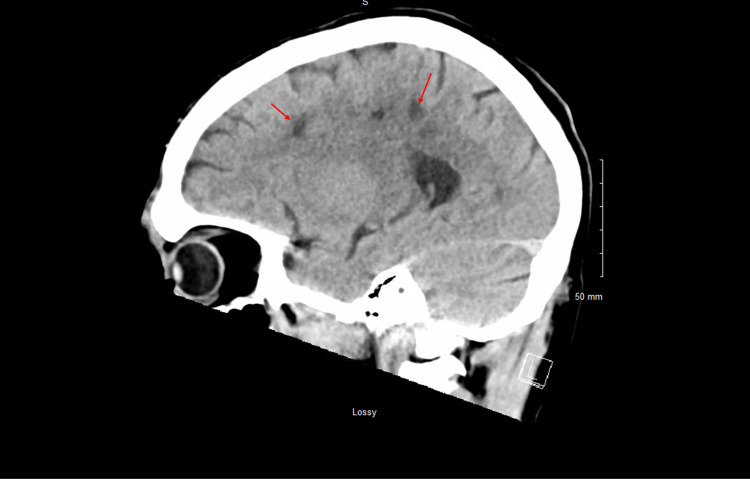
CT head sagittal view plain revealed multiple white matter hypodensities concerning septic emboli (Red arrows).

Repeat CT head, CTP of the head, and CTA of the head and neck revealed multiple white matter hypodensities concerning septic emboli, bilateral internal carotid artery 50% calcific stenosis, and no focal perfusion defects (Figure 2,3).

MRI of the brain and TEE were deferred due to his hemodynamically unstable condition. Blood cultures returned positive for *S. constellatus*, sensitive to penicillin and vancomycin but resistant to clindamycin and erythromycin. Later in the day, the patient became progressively hypotensive, and after goals of care discussion with family, palliative care was initiated. The patient passed away on day 3 of hospitalization.

## Discussion

*S. constellatus* possess multiple virulence attributes such as adhesins, fibronectin-binding ability, hydrolytic enzymes (facilitate spread), superantigens, and a polysaccharide capsule accounting for lesser chemotaxis [8,9]. Some *S. milleri* strains can attach to fibrinogen, fibrin, fibrin clot, and platelets forming a septic thrombus/vegetation [10]. The polysaccharide capsule avoids neutrophilic phagocytosis due to capsular variation [9]. The *S. milleri* group accounts for 3% to 15% of *Streptococcal* IE cases, with *S. anginosus* being responsible for most of them, followed by *S. constellatus* [4,5]. *S. constellatus* less frequently causes bacteremia and vegetation than *S. anginosus* [11]. Primary mural IE is a rare clinical presentation with the vegetation often localized in the left ventricle, and *S. aureus* is the most common cause. [12]. Primary imaging modalities to diagnose mural IE are TTE and TEE [13]. Cardiac MRI can differentiate late gadolinium enhancement (LGE) in intracardiac thrombus from the vegetation. With vegetation, endocardial LGE and a marginal mass enhancement are seen. With thrombus, phase-sensitive inversion recovery sequences reveal an increased central intensity at the lower inversion time, an etched appearance at moderate inversion time, and a decreased intensity at a longer time [14]. Patients with *S. constellatus* bacteremia need to be worked up for possible abscess collections with imaging of the abdomen, chest, and brain. Often the initial presentation is fever in 80% and a new murmur in 48% of cases [15]. The brain is frequently involved in acute IE due to the dissemination of the cardiac vegetation as septic emboli to both hemispheres [15]. MRI brain is highly sensitive in detecting the septic emboli lesions compared to CT head [15]. Mortality of 18.4% is seen in native valve IE patients with septic embolization, and two significant predictors are vegetation mobility and length >15 mm [16,17]. Individuals with immunosuppression, diabetes, cirrhosis and malignancy are at a high risk to have *S. constellatus* infection [18,19]. Indistinguishable clinical features delay diagnosis and treatment, resulting in IE progression to death in a few weeks [3]. *S. milleri* strains are usually susceptible to penicillin and cephalosporins, with a risk of penicillin resistance being less than 2%. Due to synergy with a beta-lactam agent, aminoglycosides are recommended for at least the first 2 weeks of the 4 to 6-week course of therapy with penicillin or ceftriaxone in IE [8,16]. Other than the targeted antimicrobial therapy, the surgical approach for mural IE is unknown; however, early surgery is recommended when it occurs along with valvular involvement [12].

Our patient presented with *S. constellatus* primary mural IE, a rare cause, and clinical presentation was similar to an acute stroke. He had high-grade bacteremia, and a TTE revealed a left ventricle apical vegetation. Anticoagulation absence before cardioversion increased left atrial thromboembolism risk in our patient. However, this risk is less than 2% in patients without anticoagulation [20]. He had acute encephalopathy due to multiple brain septic emboli. The possible source of his septic emboli is the cardiac vegetation (septic thrombus) localized at the left ventricular apex. He fulfilled the two major modified Duke criteria required to diagnose IE [16]. He suffered from refractory septic shock, cardiogenic shock, and atrial fibrillation. Unfortunately, he was not stable enough to get a TEE and an MRI brain. An interesting feature observed in our patient was the severity of his acute clinical presentation. *S. constellatus* virulent attributes and patient’s risk factors (IV drug abuse, poorly controlled diabetes) may be the reason for this severe presentation [2,8,18,19].

## Conclusions

This case report highlights the importance of ruling in or out infective endocarditis with a TTE or TEE once high-grade bacteremia is observed in a septic patient. Appropriate antimicrobials must be used based on local antimicrobial susceptibilities and avoid MRSA empirical coverage with clindamycin due to increased resistance in *Streptococca*l species. It also reveals the limited clinical knowledge of *S. constellatus* IE presentation. If possible, attempts to identify, stratify, and reduce immunosuppression or alter the risk factor to improve clinical outcomes should be undertaken. Although rare, the severity of the acute presentation presents further research needs in identifying the pathophysiological processes to prevent the mortality and morbidity of *S. constellatus* IE.
